# Analysis of the Occurrence of *PIK3CA* Gene Mutation in Children with Lymphatic Malformation—Single Center Study

**DOI:** 10.3390/children12111460

**Published:** 2025-10-28

**Authors:** Justyna Kukulska, Elżbieta Sałacińska-Łoś, Ewelina Perdas, Przemysław Przewratil

**Affiliations:** 1 Department of Pediatric Surgery and Oncology, Medical University of Lodz, 90-419 Lodz, Poland; elzbieta.salacinska-los@umed.lodz.pl (E.S.-Ł.); przemyslaw.przewratil@umed.lodz.pl (P.P.); 2 Department of Biostatistics and Translational Medicine, Medical University of Lodz, 90-419 Lodz, Poland; ewelina.perdas@umed.lodz.pl

**Keywords:** lymphatic malformation, *PIK3CA* mutation, targeted therapy, pediatrics, vascular malformation

## Abstract

**Highlights:**

**What are the main findings?**

- *PIK3CA* mutations were detected in 50% of children with lymphatic malformations, with the p.E542K variant being the most prevalent hotspot mutation.
- *PIK3CA* mutations were more frequently detected in isolated lymphatic malformations (63%) than in complex forms and were most commonly associated with truncal lesions.

**What is the implication of the main finding?**

- The results confirm the key role of *PIK3CA* mutations in lymphatic malformation pathogenesis and highlights the clinical importance of incorporating molecular diagnostics into routine evaluation.
- These findings provide a rationale for targeted therapies acting on the PI3K/AKT/mTOR pathway.

**Abstract:**

**Background**: Lymphatic malformations (LM) are rare congenital vascular anomalies caused by abnormal development and growth of lymphatic vessels. These malformations can lead to a wide range of symptoms, from mild swelling to more severe complications. Treatment options remain limited, especially for complex cases. Recent research has suggested that *PIK3CA* mutations play a key role in the pathogenesis of LM, potentially offering new possibilities for targeted treatment strategies. **Methods**: In this study, a cohort of 36 patients diagnosed with LM, Klippel-Trenaunay syndrome (KTS), and Proteus syndrome was analyzed. *PIK3CA* mutations were assessed in tissue samples obtained from the LM during clinically indicated procedures using digital droplet polymerase chain reaction (ddPCR), targeting five hotspots. **Results**: *PIK3CA* mutations were found in 18 patients (50%). The most frequent mutation was p.E542K (*c.1624G>A*), found in 19.44% of patients, followed by p.H1047R (*c.3149A>G*), p.E545K (*c.1633G>A*), and p.H1047L (*c.3140A>T*) each occurring in 11.11% of the cases. Mutations were more common in isolated LMs, with 63.16% of patients exhibiting *PIK3CA* mutations. **Conclusions**: *PIK3CA* mutations are common in LM, supporting the potential for targeted therapies like PI3K inhibitors in treating complex cases. This research highlights the importance of genetic analysis in the management of LM and offers a new therapeutic approach.

## 1. Introduction

Lymphatic malformations (LMs) are rare congenital anomalies, occurring in approximately 0.01% of the population, and result from developmental disorders of the lymphatic system [1,2]. They can occur in any part of the body and are classified as macrocystic (cysts ≥ 2 cm), microcystic (cysts < 2 cm), or mixed, depending on the size of the fluid-filled cysts that compose them [3,4,5]. The clinical presentation of LMs varies widely, ranging from isolated lesions (a single lump) to complex malformations associated with regional overgrowth, additional vascular anomalies, or chyle leakage [5,6]. LMs may also occur in association with other vascular malformations as part of overgrowth syndromes [7], including CLOVES (congenital lipomatous overgrowth with vascular, epidermal, and skeletal anomalies), KTS, CLAPO syndrome (capillary malformation of the lower lip, lymphatic malformation of the face and neck, asymmetry of face and limbs, and partial or generalized overgrowth), and Proteus syndrome [8].

Although LMs are histologically benign, they can significantly impair quality of life due to deformity, pain, recurrent infection, and lymphatic leakage [3,9]. Depending on their size and location, they may even be life-threatening because of swelling and obstruction of vital structures [3,10]. LMs often enlarge progressively and may increase in size during infections.

Treatment options vary according to the lesion’s location, size, and associated symptoms. Local interventions such as sclerotherapy, compression therapy, laser therapy, and surgery are often insufficient for complex malformations [1,5], which underscores the ongoing need for effective targeted systemic therapies.

Over the years, research has shown that LMs and overgrowth syndromes have heterogeneous etiologies, which vary according to their clinical presentation. Lymphatic cysts are lined by lymphatic endothelial cells (LECs) that express characteristic lymphatic markers [11]. Vascular endothelial growth factor C (VEGF-C) induces lymphangiogenesis through binding to VEGF receptor 3 (VEGFR-3), which is predominantly expressed on LECs. In *PIK3CA*-driven LMs, VEGFR-3 expression is abnormally elevated in LECs, linking PI3K hyperactivation with enhanced VEGF-C signaling and pathological lymphangiogenesis [12].

Beyond the PI3K/AKT/mTOR pathway, other signaling cascades also play important roles in the pathogenesis of complex lymphatic anomalies (CLAs). Disorders such as Gorham-Stout disease (GSD), kaposiform lymphangiomatosis (KLA), central conducting lymphatic anomaly (CCLA), and generalized lymphatic anomaly (GLA) have been linked to alterations in the RAS/RAF/MEK/ERK signaling pathway [5,13]. RASopathies encompass vascular anomalies driven by dysregulation of this pathway. The MAPK pathway regulates essential cellular processes, including differentiation, proliferation, apoptosis, and responses to cellular stress. Upon stimulation by growth factors or cytokines, RAS activation triggers a sequential phosphorylation cascade through RAF, MEK, and ERK, with activated ERK translocating to the nucleus to regulate gene expression. Aberrations in this pathway have been identified in several vascular malformations [5,13]. Importantly, crosstalk between the RAS/ERK and PI3K/AKT pathways underscores their interconnected roles in the pathogenesis of vascular anomalies [5].

Numerous studies have demonstrated that LMs are most frequently associated with postzygotic, somatic activating mutations in the phosphatidylinositol-4,5-bisphosphate 3-kinase catalytic subunit alpha (*PIK3CA*) gene [1,5,10,11,12]. *PIK3CA* encodes the catalytic subunit of phosphoinositide 3-kinase (PI3K), a key regulator of cellular growth, survival, and metabolism [13]. Activating mutations lead to hyperactivation of the PI3K/AKT/mTOR signaling pathway, which involves multiple downstream effectors such as AKT and mTOR, thereby promoting vascular malformations and abnormal tissue overgrowth [12,14,15]. Pathogenic *PIK3CA* variants have been identified not only in non-neoplastic overgrowth syndromes but also across a variety of cancer types. Among non-cancerous disorders, *PIK3CA* mutations define the *PIK3CA*-Related Overgrowth Spectrum (PROS), encompassing a wide range of conditions characterized by segmental tissue overgrowth and vascular malformations, including CLOVES syndrome, KTS, CLAPO syndrome, and fibro-adipose vascular anomaly [5]. In oncology, *PIK3CA* mutations represent one of the most common oncogenic alterations, particularly in breast, colorectal, endometrial, ovarian, cervical, urothelial, and head and neck squamous cell carcinoma [15,16,17,18].

The identification of these mutations holds significant therapeutic potential. Targeting the PI3K/AKT/mTOR signaling pathway currently represents the most advanced treatment strategy [4,5]. Sirolimus is widely used, including in our department, with encouraging clinical outcomes [3,5,6,9]. Another promising agent is alpelisib, a PI3K-specific inhibitor that is currently under investigation [19,20,21]. Miransertib, an AKT inhibitor, is also in development; however, information regarding its efficacy in the treatment of LMs is not yet available. Additionally, disruption of the RAS/MAPK pathway—primarily implicated in CLAs—may be targeted using the MEK inhibitor trametinib, although clinical data in this context remain also very limited [12].

In our study, we analyzed *PIK3CA* mutations at several critical hotspots that have been implicated in various vascular malformations, including those affecting the lymphatic system [5]. The five targeted hotspots were p.E542K (*c.1624G>A*), p.H1047R (*c.3140A>G*), p.E545K (*c.1633G>A*), p.H1047L (*c.3140A>T*), and p.E545G (*c.1634A>G*). These mutations are believed to contribute to the pathogenesis of LMs by enhancing lymphangiogenesis and promoting the abnormal proliferation and overgrowth of lymphatic vessels.

## 2. Materials and Methods

### 2.1. Patient Cohort and Diagnostic Criteria

The study included a cohort of 36 patients recruited from the Department of Pediatric Surgery and Oncology, Central Teaching Hospital, Lodz, between 2010 and 2022. Patients’ characteristics are presented in Table 1. This represents the largest cohort of patients with LMs in Poland in whom the occurrence of *PIK3CA* gene mutations has been systematically analyzed.

All the patients were under 18 years of age at the time of surgery or biopsy, and all were diagnosed with LM, Proteus syndrome, or KTS. The diagnosis of LM was histopathologically confirmed in all cases based on the analysis of tissue samples obtained during clinically indicated surgery or biopsy. Proteus syndrome was diagnosed based on the presence of characteristic features, including disproportionate and asymmetric overgrowth of body parts (particularly involving the skeleton), epidermal nevi, vascular malformations, and dysregulated adipose tissue [14,22]. The diagnosis of KTS was established based on coexistence of LM with capillary malformation (CM), limb hypertrophy, and venous malformation (VM) [11,13]. Patients with previously confirmed *PIK3CA* mutations or histopathological findings of mixed vascular malformations were excluded from the study.

Data on the clinical characteristics of the patients, including age at biopsy or surgery, gender, type of LM (isolated vs. complex), lesion location (head-neck, truncal, limb), and associated syndromes (KTS, Proteus syndrome), were collected from patients’ medical records. Diagnostic imaging results (e.g., ultrasound, MRI) were used to supplement clinical data.

### 2.2. Tissue Samples

The study was conducted on tissue samples obtained from the LMs. Prospectively, tissue samples were collected during clinically indicated surgeries or biopsies and subsequently formalin-fixed and paraffin-embedded (FFPE). Retrospectively, samples were retrieved from archived FFPE tissue blocks. All collected specimens underwent histopathological analysis.

### 2.3. Genetic Analysis

The search for mutations was performed using ddPCR method, which is well suited for the detection of rare genetic variants. This technique provides high sensitivity and specificity [8,11,15]. DNA was extracted from the FFPE tissue samples using QIAamp DNA FFPE Tissue Kit (Qiagen, Germantown, MD, USA) [23]. This is a method optimized for DNA isolation from FFPE samples. The finely cut tissue fragments, embedded in paraffin, were subjected to the deparaffinization process using Deparaffinization Solution (Qiagen). After adding 160 μL of Deparaffinization Solution to the samples, mixing thoroughly, and centrifuging, the material was incubated at 56 °C for 3 min and then left at 20 °C until completely cooled. Next, 180 μL of ATL buffer was added, and the samples were centrifuged at 11,000× *g* for 1 min. In the next step, 20 μL of proteinase K was added to the lower, clear phase. The mixture was incubated at 56 °C for 1 h. After incubation and a brief centrifugation, the lower, clear phase was transferred to new 2 mL tubes for DNA isolation. DNA isolation was performed according to the manufacturer’s protocol (QIAamp DNA FFPE Tissue Kit; Qiagen) using buffers AL, AW1, and AW2. The DNA was eluted in 50 μL of ATE buffer. The quantitative analysis of DNA was performed using the Picodrop spectrophotometer (Picodrop, Hinxton, UK). Then, a ddPCR assay was used to detect mutations in the *PIK3CA* gene. The assay targeted specific hotspots within the *PIK3CA* gene, known for their possible association with causing LMs. The five targeted hotspots were *PIK3CA* p.H1047R, *PIK3CA* p.H1047L, *PIK3CA* p.E545K, *PIK3CA* p.E542K, *PIK3CA* p.E545G. The study used 11 μL of ddPCR Supermix of Probes (without dUTP, Bio-Rad, Hercules, CA, USA), 1.1 μL of ddPCR Mutant Assay *PIK3CA* (Bio-Rad), and 5 ng of DNA. The reaction was performed in a total volume of 20 μL. Water was used as a ‘no template control’ (NTC). Droplets were generated using QX 200 droplet generator (Bio-Rad) in an 8-well plate. The plate contained 20 μL of the reaction mixture and 70 μL of oil. A total of 40 μL of the prepared droplet mixture was transferred to a 96-well plate. The PCR reaction was conducted in a T100 Thermal Cycler (Bio-Rad). The PCR was conducted using following parameters: 1 cycle (10 min) at 95 °C, then 40 cycles of 30 s at 94 °C, and 40 cycles of 60 s at 55 °C, followed by 1 cycle (10 min) at 98 °C, and then kept at 4 °C.

### 2.4. Statistical Analysis

According to the manufacturer’s validation data and best practice guidelines, the ddPCR assay used in this study has a sensitivity of approximately 1% variant allele frequency (VAF), allowing for reliable detection of low-frequency mosaic variants. Samples with three or more positive mutant droplets were considered positive, in accordance with best practice guidelines for rare mutation detection [24].

The Shapiro–Wilk test was performed to test for normal distribution. Continuous variables are presented as medians with the values of the lower and upper quartiles (25–75th percentiles). Categorical variables are presented as numbers with an appropriate percentage. Analysis of the differences between groups for nominal variables was carried out using 2 × 2 tables, the significance of which was verified by Chi2, Chi2 with Yates correction, or Fisher’s exact test, as appropriate.

### 2.5. Ethical Approval

The study received ethical approval from the Bioethical Committee at the Medical University of Lodz (No. RNN/55/22/KE).

## 3. Results

The study cohort comprised 36 patients diagnosed with LM and associated syndromes. The median age at the time of biopsy or surgical resection was 3 years (range: 0.5–17 years) (Table 1). The cohort included 24 females (66.67%) and 12 males (33.33%), all of whom were under 18 years of age at the time of the procedure. In total, 19 patients (52.78%) had isolated LMs, whereas 17 patients (47.22%) presented with complex LMs.

The anatomical distribution of LMs was as follows: 18 patients (50%) had truncal LMs, 12 patients (33.33%) had head and neck LMs, 4 (11.11%) had limb LMs, 1 (2.78%) had both limb and truncal LMs, and 1 (2.78%) had LMs involving the head and neck, limb, and truncal regions.

*PIK3CA* mutations were identified in 18 patients (50%) using ddPCR. The assay targeted specific hotspots within the *PIK3CA* gene, and samples were considered positive if they contained three or more positive mutant droplets. ddPCR data were analyzed using QuantaSoft software (version 1.7.4.0917), and only samples with more than 10,000 droplets were included in the analysis.

**Figure 1:**
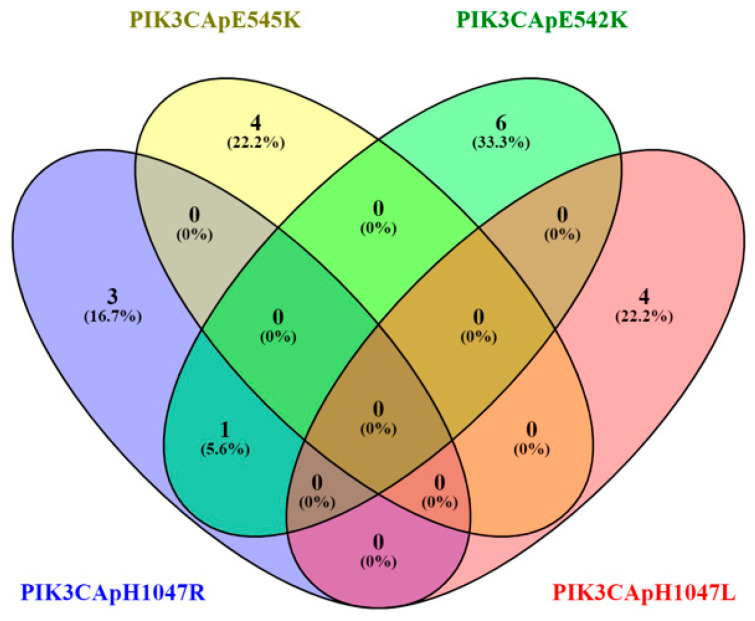
Venn diagram of *PIK3CA* mutations hotspots.

Five known *PIK3CA* hotspots were examined. Among the mutation-positive cases, p.E542K (*c.1624G>A*) was the most frequent variant, detected in 7 patients (19.44%). The p.H1047R (*c.3140A>G*), p.E545K (*c.1633G>A*), and p.H1047L (*c.3140A>T*) variants were each identified in 4 patients (11.11%); while p.E545G (*c.1634A>G*) was not detected in any sample (shown in Figure 1).

Within the mutated lesions, the VAF ranged from 1.1 to 12%, with a median VAF of 5.0%. Among the 18 patients harboring *PIK3CA* mutations, most exhibited a mutation in a single hotspot—17 patients (47.22%)—whereas 1 patient (2.78%) carried mutations in two distinct hotspots.

**Figure 2:**
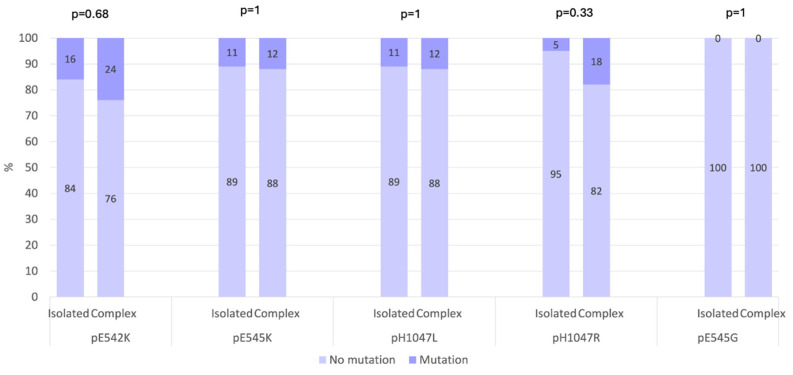
Prevalence of gene mutations in hotspots across isolated and complex LMs (*p*-value Fisher’s exact test).

When examining the type of LM, a higher frequency of *PIK3CA* mutations was observed in patients with isolated LMs (12 of 19; 63.16%) compared with those with complex LMs (6 of 17; 35.29%) (Figure 2). In our cohort, the distribution of *PIK3CA* mutations according to the anatomical location of the LM showed that truncal LMs were most commonly associated with mutations, occurring in 10 of 18 patients (55.56%). Mutations were also detected in 4 of 18 patients (22.22%) with head and neck LMs and in 2 of 18 patients (11.11%) with limb LMs. In addition, one patient (5.56%) with both limb and truncal LMs and one patient (5.56%) with head and neck, limb and truncal LMs carried *PIK3CA* mutations. Overall, these findings indicated that *PIK3CA* mutations were most frequently found in truncal LMs, although they also occurred in head and neck and limb locations.

Among the patients who underwent genetic testing for *PIK3CA* mutations, seven individuals with confirmed mutations were subsequently treated with the mTOR inhibitor sirolimus as part of their clinical management. Other targeted agents, such as PI3K or AKT inhibitors, have not yet been administered to our patients, as additional ethical approval is required and has not yet been obtained. Although treatment information was available in the clinical records, analysis of therapeutic outcomes was beyond the scope of the present study and is therefore not reported here.

## 4. Discussion

LMs are rare congenital vascular anomalies that result from abnormal development of the lymphatic vessels [1,5]. Their clinical presentation ranges from localized lesions to extensive, complex forms that can cause functional impairment, and significant morbidity. In this study, we analyzed the occurrence of *PIK3CA* mutations in 36 pediatric patients with LMs, representing the largest cohort reported from Poland to date.

Using ddPCR, *PIK3CA* mutations were identified in 50% of patients. Although this detection rate is somewhat lower than reported in several other studies, it nevertheless supports the association of *PIK3CA* mutations with the pathogenesis of LMs [10,11,12]. The most frequent variant was *PIK3CA* p.E542K (*c.1624G>A*), followed by p.H1047R (*c.3140A>G*), p.E545K (*c.1633G>A*), and p.H1047L (*c.3140A>T*). These findings are consistent with previous studies that report similar hotspot distributions in patients with isolated or syndromic LMs [12,17]. Mutations occurred more frequently in isolated than in complex LMs and were most often detected in truncal lesions. This distribution suggests possible genotype-phenotype correlations, although further studies in larger, multicenter cohorts are needed to validate these associations.

The identification of *PIK3CA* mutations provides a molecular basis for the use of targeted therapies that inhibit the PI3K/AKT/mTOR signaling pathway [1,4,5,12]. In our cohort, several patients with confirmed mutations were treated with sirolimus, an mTOR inhibitor shown to reduce lesion size and improve clinical symptoms in both microcystic and macrocystic forms. Other emerging agents, such as PI3K inhibitor alpelisib, have shown encouraging early clinical results and may represent future therapeutic options for refractory or complex cases [25]. Another agent, miransertib, an AKT inhibitor, is under investigation for low-flow vascular malformations [26], although data specifically addressing its effects in the LMs are not yet available [12]. Although treatment outcomes were not assessed in this study, the identification of activating *PIK3CA* variants highlights the potential for genotype-guided therapy and supports the incorporation of molecular diagnostics into clinical management.

While ddPCR proved highly effective for detecting hotspot variants, its scope is limited to predefined regions of the *PIK3CA* gene. Broader genomic approaches, such as next-generation sequencing (NGS), can detect non-hotspot variants and additional genes implicated in vascular anomalies, providing a more comprehensive genetic profile [13]. Expanding molecular diagnostics in this direction may improve mutation detection rates and support individualized treatment strategies.

Several limitations of our study should be acknowledged. It was conducted at a single center, and larger multicenter investigations are needed to confirm the prevalence of *PIK3CA* mutations and their clinical correlations. Although ddPCR is a highly sensitive and specific technique for mutation detection, it is restricted to known hotspots. Nevertheless, this method offers important advantages: it enables the detection of low-frequency variants in mosaic samples, is cost-effective compared with NGS, and is particularly suitable for DNA extracted from formalin-fixed paraffin-embedded (FFPE) tissues—the most common material available for retrospective studies. Moreover, ddPCR has been widely validated in vascular anomaly research and remains a reliable approach for mutation analysis [7,8,11,15,26,27].

In summary, our findings confirm that *PIK3CA* mutations are common in lymphatic malformations and play a pivotal role in their pathogenesis. Genetic testing should be considered an integral component of the diagnostic evaluation, as it may guide targeted therapeutic decisions. Further research using broader genomic methods and larger patient cohorts will be essential to refine genotype-phenotype correlations and optimize personalized treatment approaches for pediatric patients with LMs.

## 5. Conclusions

In conclusion, this study confirms that *PIK3CA* mutations play a key role in the pathogenesis of LMs. Identifying these variants enhances understanding of LM biology and supports the integration of genetic testing into clinical practice. Targeted therapies acting on the PI3K/AKT/mTOR pathway, such as sirolimus or alpelisib, offer promising treatment options for affected children, though further studies are needed to establish their long-term safety and efficacy. Future research using broader genomic approaches, including NGS, will be essential to detect non-hotspot variants and refine personalized treatment strategies for pediatric patients with LMs.

## Figures and Tables

**Table 1:** Patient characteristics.

Patient	Age at the Biopsy or Surgery (Years)	Gender	Diagnosis	Location	Type of LM	Detected Mutation in *PIK3CA*	VAF (%)
1	7	F	LM, Proteus syndrome	truncal	complex	p.E542K	7
2	2	F	LM	truncal	isolated	-	-
3	1	M	LM	upper limb	complex	p.H1047R	12
4	1.75	F	LM	truncal	complex	p.H1047L	6.6
5	2	F	LM	head-neck	complex	p.E545K	9
6	3	M	LM, Proteus syndrome	Head-neck, truncal, limb	complex	p.E545K	7
7	4	F	LM, KTS	lower limb, truncal	complex	p.H1047R	3
8	2	F	LM	truncal	complex	p.E542K	3.3
9	17	F	LM	lower limb	isolated	-	-
10	2	M	LM	truncal	complex	p.E542K	1.2
11	2	F	LM	head-neck	complex	p.H1047R	3.9
12	5	F	LM	truncal	isolated	p.E545K	4.4
13	5	F	LM	head-neck	isolated	p.E542K, p.H1047R	2.2 7.7
14	7	F	LM	truncal	isolated	p.E542K	8
15	3	M	LM	head-neck	complex	-	-
16	1.08	F	LM	head-neck	complex	-	-
17	1.25	F	LM	truncal	complex	p.E542K	5.5
18	12	F	LM	head-neck	isolated	-	-
19	1	M	LM	truncal	complex	-	-
20	2	F	LM	upper limb	isolated	p.E542K	4.8
21	3	M	LM	head-neck	complex	p.H1047L	3.9
22	3	F	LM	truncal	complex	-	-
23	0.5	F	LM	truncal	isolated	p.E545K	1.1
24	4	M	LM	truncal	isolated	-	-
25	17	F	LM	head-neck	isolated	-	-
26	14	M	LM	head-neck	complex	-	-
27	1	F	LM	truncal	isolated	p.H1047L	5.9
28	8	F	LM	head-neck	isolated	-	-
29	3	M	LM	truncal	isolated	-	-
30	10	F	LM	lower limb	isolated	-	-
31	16	F	LM	head-neck	complex	-	-
32	5	M	LM	head-neck	isolated	-	-
33	2	M	LM	truncal	isolated	-	-
34	1.4	M	LM	truncal	isolated	-	-
35	1	F	LM	truncal	isolated	p.H1047L	5
36	2	F	LM	truncal	isolated	-	-

## Data Availability

The original contributions presented in this study are included in the article/Supplementary Materials. Further inquiries can be directed to the corresponding author.

## Supplementary Materials

The following supporting information can be downloaded at: https://www.mdpi.com/article/10.3390/children12111460/s1, Supplementary Table S1. Quantitative results of droplet digital PCR (ddPCR) analysis for PIK3CA hotspot mutations (Each worksheet presents the quantitative ddPCR output for a specific PIK3CA hotspot mutation. Data include sample identifiers, experiment type, concentration, variant allele frequency, and Poisson-based estimations). Supplementary Table S2. The ddPCR assays used for detection of PIK3CA hotspot mutations.

## Abbreviations

The following abbreviations are used in this manuscript:

LM	Lymphatic malformation
PIK3CA	Phosphatidylinositol-4,5-bisphosphate 3-kinase, catalytic subunit alpha
KTS	Klippel-Trenaunay syndrome
ddPCR	Digital droplet polymerase chain reaction
PI3K	Phosphoinositide 3-Kinase
CLOVES	Congenital lipomatous overgrowth with vascular, epidermal, and skeletal anomalies
CLAPO	Capillary malformation of the lower lip, lymphatic malformation of the face and neck, asymmetry of face and limbs, partial/generalized overgrowth
mTOR	Mechanistic target of rapamycin
VAF	variant allele frequency
CM	Capillary malformation
VM	Venous malformation
FFPE	Formalin-fixed paraffin embedded
NTC	No template control
MRI	Magnetic resonance imaging
NGS	Next-generation sequencing
